# P-549. Early Results from a Community-Based HIV Rapid Start Initiative, Memphis TN

**DOI:** 10.1093/ofid/ofae631.748

**Published:** 2025-01-29

**Authors:** David Closs

**Affiliations:** Friends For All Memphis, Memphis, Tennessee

## Abstract

**Background:**

In 2023, Memphis ranked second in the US for the highest rates of new HIV infections. Friends for All (FFA) is a community-based organization located in Memphis whose aim is to prevent the spread of HIV and improve health outcomes of those living with HIV through a holistic and client-centered approach. To improve health outcomes for people diagnosed with HIV and prevent further transmission, FFA launched a rapid test and treat HIV initiative with the primary aim of increasing viral suppression and retention in care.

**Methods:**

We offered free HIV testing at our sexual health clinic and pop-up community testing sites. Individuals that tested positive for HIV and had no prior HIV diagnosis were offered rapid start HIV treatment, defined as treatment initiation within 72 hours. Staff administered an assessment of barriers to linkage and scheduled the appointment.

**Results:**

Between July 2023 and March 2024, FFA tested 2131 clients for HIV. Of the 125 who tested positive, 14 met rapid start criteria. We successfully initiated treatment inside the 72-hour window for 12 (83%) of the 14 eligible. Ninety days post rapid start, 10 (83%) achieved viral suppression and 10 (83%) attended at least 3 medical appointments. The two clients who did not initiate rapid start treatment were identified at pop-up events outside of normal clinic hours, and it was not possible to schedule an appointment within 72 hours. Both of these clients did, however, initiate treatment within 7 days of diagnosis.

**Conclusion:**

While linkage to rapid start ART has been successful in the clinical setting, barriers remain at the patient and organizational level that prevent similar outcomes in the outreach setting. Patients reported barriers to appointment attendance, including food insecurity and transportation. At the organizational level, limited access to ART outside of clinic hours and prescriber requirements for in-person care prevent successful linkage from outreach settings. We are investigating the use of a mobile unit to initiate rapid start treatment in outreach settings and telehealth options for ongoing HIV care. To address patient level barriers, FFA will continue to provide transportation and food services to those living with HIV.

**Disclosures:**

**David Closs, MPH**, Gilead Sciences: Grant/Research Support

